# Purification and Characterization of a Fibrinolytic Enzyme from Marine *Bacillus velezensis* Z01 and Assessment of Its Therapeutic Efficacy In Vivo

**DOI:** 10.3390/microorganisms10050843

**Published:** 2022-04-20

**Authors:** Yuting Zhou, Huizhen Chen, Bo Yu, Guiguang Chen, Zhiqun Liang

**Affiliations:** State Key Laboratory for Conservation and Utilization of Subtropical Agro-Bioresources, Guangxi Microorganism and Enzyme Research Center of Engineering Technology, College of Life Science and Technology, Guangxi University, 100 Daxue Road, Nanning 530004, China; 1808401025@st.gxu.edu.cn (Y.Z.); 2008391001@st.gxu.edu.cn (H.C.); 1908401010@st.gxu.edu.cn (B.Y.)

**Keywords:** fibrinolytic enzyme, antiplatelet, anticoagulation, thrombolytic

## Abstract

Fibrinolytic enzymes are the most effective agents for the treatment of thrombotic diseases. In the present study, we purified and characterized an extracellular fibrinolytic serine metalloprotease (named Velefibrinase) that is produced by marine *Bacillus velezensis* Z01 and assessed its thrombolysis in vivo. SDS-PAGE and MALDI-TOF-MS analyses showed that the molecular mass of Velefibrinase was 32.3 KDa and belonged to the peptidase S8 family. The optimal fibrinolytic activity conditions of Velefibrinase were 40 °C and pH 7.0. Moreover, Velefibrinase exhibited high substrate specificity to fibrin, and a higher ratio of fibrinolytic/caseinolytic (1.48) values, which indicated that Velefibrinase had excellent fibrinolytic properties. Based on the degradation pattern of fibrin and fibrinogen, Velefibrinase could be classified as α/β-fibrinogenase. In vitro, Velefibrinase demonstrated efficient thrombolytic ability, anti-platelet aggregation, and amelioration of blood coagulation (APTT, PT, TT, and FIB), which were superior to those of commercial anticoagulant urokinase. Velefibrinase showed no hemolysis for erythrocyte in vitro and no hemorrhagic activity in vivo. Finally, Velefibrinase effectively prevented mouse tail thrombosis in a dose-dependent (0.22–0.88 mg/kg) manner. These findings suggested that Velefibrinase has the potential to becoming a new thrombolytic agent.

## 1. Introduction

The accumulation of insoluble fibrin in a blood vessel usually leads to thrombosis, which further result in thrombotic diseases (TDs), such as myocardial infarction, stroke, and venous thromboembolism [1]. TDs account for approximately 31% of all worldwide deaths and are rapidly increasing in developing countries [2]. The imbalance between coagulation and fibrinolysis is one of the essential pathophysiological processes of TDs [3]. Under pathophysiological conditions, owing to the overproduction of thrombin and uncontrolled hydrolysis from fibrinogen (FIB) to fibrin, insoluble fibrin fibers can precipitate in the blood vessel, which poses a clinical challenge for treatment [4]. Therefore, there is a significant demand for thrombolytic agents to treat patients with TDs.

Fibrinolytic enzymes are proteases responsible for degrading fibrin in thrombus. Based on their mechanism, fibrinolytic enzymes can be divided into two types: One is plasminogen activators, such as tissue type plasminogen activator (t-PA) and urokinase, which can hydrolytic plasminogen into plasmin; the other is fibrin(ogen)olytic enzyme, such as nattokinase and lumbrokinase [5]. Recently, several fibrinolytic enzymes have been applied clinically as thrombolytic agents. However, these agents have limitations in clinical settings, such as the high cost, short half-life, and side effects, which include excessive bleeding and low specificity for fibrin [6,7]. Therefore, significant research efforts have been devoted to identifying more effective and specific fibrinolytic agents from natural sources, especially marine environments.

Although certain fibrinolytic enzymes from marine environments have been purified and characterized, the vast diversity of marine resources can be exploited further [8]. Given that marine organisms are exposed to extreme environments of high pressure, temperature, and salinity over a prolonged period, enzymes produced by marine microorganisms likely have advantages over those produced by terrestrial microorganisms [9]. Moreover, it is believed that the saline content of seawater in natural and chemically is very similar to human blood plasma, thus these enzymes may have lower or even no toxicity and side effects when used for thrombus therapy [10]. Therefore, the marine environment will have strong potential to discover novel fibrinolytic enzymes with unique biochemical properties.

*Bacillus velezensis* was found on the river-bank of Vélez in Málaga, Spain in 2005 and produces various metabolic intermediates, such as antibiotics, antioxidants, antifungal factors, and growth promoters [11,12]. Data on *Bacillus velezensis* have been applied widely to the field of biological control and promotion of plant growth [13] and have shown its potential as a probiotic [14]. However, several studies only reported the discovery of and cloned fibrinolytic enzyme in *Bacillus velezensis* [15,16,17,18], therefore, it is a topic of further research. In the present study, a fibrinolytic protease (named Velefibrinase) was purified from marine *B. velezensis* Z01, which was screened from sea mud and its biochemical property was characterized. In addition to determining the thrombolytic pattern and the antiplatelet and anticoagulation properties of Velefibrinase, we evaluated the safety of its clinical application. Finally, the therapeutic potency of Velefibrinase was tested on in a rodent animal model.

## 2. Materials and Methods

### 2.1. Materials

A quantitative protein kit (Bradford) was purchased from TransGen Biotech (Beijing, China). Thrombin and urokinase standards were provided by Hunan Yige Pharmaceutical Co., Ltd. (Xiangtan, China) and the National Institutes for Food and Drug Control (Beijing, China), respectively. Bovine serum albumin (BSA), FIB, adenosine diphosphate (ADP), ethylene diamine tetraacetic acid (EDTA), phenylmethanesulfonyl fluoride (PMSF), pepstatin A, and β-mercaptoethanol were purchased from Sigma-Aldrich (St. Louis, MO, USA). All other chemicals were of analytical or sequencing grade.

### 2.2. Isolation and Taxonomic Identification of Fibrinolytic Enzyme-Producing Marine Microorganisms

The isolation and identification of marine microorganism strains with the fibrinolytic enzyme were performed following previously described procedures [19]. Briefly, the sea mud (obtained from various places in Beibu Gulf, South China Sea) was continuously diluted to 10^−6^ with 0.02 M phosphate buffer saline (PBS, pH 7.2) and plated on fibrin agar plates (pH 7.2). The plates were incubated at 37 °C for 48 h, and the fibrinolytic potency was evaluated by the diameter ratio of transparent circle to bacterial colony. The promising strains were selected according to the diameter ratio. From the above results, one potential bacterial strain with the highest fibrinolytic to caseinolytic (F/C) ratio was selected for further study. The taxonomic identification of the potential strain was performed by (a) biochemical properties and phenotypic characterization; (b) sequencing of the 16S rRNA, *gyrB* and *rpoB* genes and phylogenetic analysis; (c) observation of cell morphology using a scanning electron microscope (SEM). The deduced nucleotide sequences were searched using BLASTn, and homologous sequences were retrieved from GenBank databases. Using the MEGA7 multiple sequence alignment program, the partial 16S rDNA, *gyrB*, and *rpoB* genes were aligned with homologous DNA sequences deposited in the NCBI databases. The phylogenetic tree was constructed using the neighbor-joining method.

### 2.3. Purification and Identification of Fibrinolytic Enzyme

Unless otherwise stated, all steps were conducted at 4 °C. The fermented broth was centrifuged at 5000 rpm for 10 min, and the cell-free supernatant was precipitated by ice-cold ethanol (60–80%, *v*/*v*) for 4 h. The precipitates were then re-suspended in 20 mM sodium phosphate buffer containing 1.9 M ammonium sulfate. The supernatant was obtained by centrifuging at 10,000 rpm for 10 min after allowing to stand for 12 h, and was filtered and concentrated in a tangential flow membrane filtration system (GE, Amersham, Westborough, MA, USA]. Subsequently, the concentrated supernatant was applied to the t-Butyl HIC column (Macro-Prep^®^, Hercules, CA, USA) pre-equilibrated with 20 mM sodium phosphate buffer containing 1.9 M ammonium sulfate, and then eluted with 20 mM sodium phosphate buffer with pH 7.5. Next, the fractions of fibrinolytic activity were pooled, dialyzed against a glycine-sodium hydroxide buffer (pH 10.0), concentrated and loaded up to a DEAE-Sephadex (Macro-Prep^®^, Hercules, CA, USA) column, and eluted with the same buffer containing 1 M sodium chloride. Finally, the active fractions were collected, desalted, lyophilized, and identified using 12% SDS-PAGE.

The secondary structure of the enzyme was determined using a circular dichroism (CD) analyzer (MOS-450, CBIO-LOGIC, France) as per a previously described method [20]. The CD spectrum of Velefibrinase was analyzed, and a secondary structure was inferred using CDPro (http://lamar.colostate.edu/~sreeram/CDPro, accessed on 12 August 2021). Moreover, an aliquot of trypsin-digested peptide fragments of purified and desalted enzymes (1.0 μg) was ascertained by MALDI-TOF-MS (4800, Applied Biosystems, Framingham, MA, USA) analysis using a synapinic acid matrix [21].

### 2.4. Evaluation of Protein Concentration and Fibrinolytic Activity

Protein concentration was measured using the Bradford method [22]. Briefly, aliquots of the 200 μL sample were added to 1 mL of Bradford working liquid, mixed, and incubated at 37 °C for 5 min. The absorbance was determined at 595 nm using a plate reader (MULTISKAN GO, Thermo Scientific, Vantaa, Finland). BSA was used as standard, according to manufacturer instructions.

Both plasminogen-free and plasminogen-rich fibrin plates were used to measure fibrinolytic activity following the method described by Astrup [23]. Subsequently, samples were carefully added into each hole, which was punched into the plate and incubated at 37 °C for 12 h. Fibrinolytic activity was evaluated by the area of the clear zone around each hole and the urokinase as a control.

The degradation patterns of FIB and fibrin were analyzed following the method described by Liu [5] with minor modifications. Briefly, 20 μL of 1% (*w*/*v*) FIB or fibrin was incubated with 10 μL (0.4 μM) Velefibrinase in 20 mM PBS (pH 7.0) at 40 °C at various times intervals (0–80 min). The reaction was terminated by adding 6 μL of denatured buffer that was boiled for 10 min, and analysis was conducted using 12% SDS-PAGE.

### 2.5. Determination of Biochemical Properties

The optimal pH of the enzyme was evaluated by measuring fibrinolytic activity at a pH range of 4.0–11.0. To determine the pH stability of Velefibrinase, the enzyme samples were added to various pH buffers (Na_2_HPO_3_-sodium citrate [pH 4.0–6.0], NaH_2_PO_4_-Na_2_HPO_4_ [pH 6.0–8.0], Tris-HCl [pH 8.0–9.0], glycine-NaOH [pH 9.0–10.0], and Na_2_CO_3_-Na_2_HCO_3_ [pH 10.0–11.0]). After incubating at 37 °C for 24 h, the residual enzyme activity was evaluated using the plasminogen-free fibrin plate method as described above. The optimum temperature for Velefibrinase was determined by measuring the activity with fibrin as a substrate and incubating with 20 mM of PBS (pH 7.0) at a temperature range of 20 to 80 °C. To observe the thermal stability of the enzyme, the residual activity was measured using the plasminogen-free fibrin plate method while incubating in the same buffer at different temperatures (20–80 °C) for 24 h. The effect of various metal ions and protein inhibitors on the fibrinolytic enzyme was assessed using CuCl_2_, NaCl_2_, MnCl_2_, CoCl_2_, ZnCl_2_, MgCl_2_, CaCl_2_, PMSF, EDTA, β-Mercaptoethanol, and Pepstatin A in 0.02 M of PBS buffer at pH 7.0.

To investigate the specificity of the protease activity of Velefibrinase against protein substrates, which include casein, FIB, fibrin, and albumin, we used the method followed by Lowry [24]. A specified amount of the enzyme was incubated with 1% (*w*/*v*) of the various substrates dissolved in 20 mM sodium phosphate buffer at pH 7.0 at 40 °C. After 10 min of incubation, the tyrosine content in the supernatant was determined using Folin–Ciocalteu’s reagent at 660 nm. One unit (U) of protease activity was defined as 1 μg tyrosine released/min/mL of enzyme at 40 °C. The Michaelis–Menten constant (*K*_m_) and maximum velocity (*V*_max_) value of the enzyme were calculated using the Lineweaver–Burk double reciprocal plot.

### 2.6. Assessment of Thrombolytic Activity In Vitro

To evaluate thrombolytic activity in vitro, 100 μL of 0.25 M CaCl_2_ was added to 1.0 mL of buffalo blood containing 3.8% sodium citrate, and allowed to coagulate at room temperature for 1 h. Aliquots of 500 μL with various concentrations (0.2–0.8 μM) of Velefibrinase were incubated with 0.1 g blood clot at 37 °C for 10 h. For the control, a clot was added to PBS under identical conditions. The following formula was used to calculate thrombolytic activity:

(1) $$\mathrm{Thrombolytic}\;\mathrm{activity}\;\left(\%\right)=\frac{\mathrm{Rest}\;\mathrm{weight}\;\mathrm{of}\;\mathrm{blood}\;\mathrm{clot}\;\mathrm{in}\;\mathrm{Velefibrinase}-\mathrm{Rest}\;\mathrm{weight}\;\mathrm{of}\;\mathrm{blood}\;\mathrm{clot}\;\mathrm{in}\;\mathrm{PBS}}{0.1-\;\mathrm{Rest}\;\mathrm{weight}\;\mathrm{of}\;\mathrm{blood}\;\mathrm{clot}\;\mathrm{in}\;\mathrm{PBS}}\times 100$$

### 2.7. Determination of Antiplatelet and Anticoagulation Activity In Vitro

The effect of Velefibrinase on ADP-induced platelet aggregation was evaluated using the method described by Xia [25]. In brief, the platelet number was determined by blood cell count and platelet-rich plasma (PRP), and wash platelet (WP) was adjusted at 3 × 10^8^ cells/mL by platelet-poor plasma (PPP) and HEPES-Tyrode’s buffer, respectively. Various concentrations (0.2–0.8 μM) of Velefibrinase and urokinase were incubated with 300 μL PRP or WP at 37 °C for 10 min. Then ADP (5 μM) was added, and platelet aggregation was recorded using an aggregometer (SC-40, STEELLEX & Co, Beijing, China). Additionally, to evaluate the maximum platelet aggregation of WP, the FIB was mixed into WP to achieve a final concentration of 0.3 g/L. For the control, the sample was induced by ADP in the absence of the enzyme.

The method used to study the anticoagulant activity of the enzyme in vitro is described in the study by Doley [26]. The PPP was prepared by centrifugation of fresh blood at 3000 rpm for 10 min at 4 °C. Various concentrations of the enzyme solution were pre-incubated with 300 μL of PPP for 10 min at 37 °C. The effect of the enzyme on activated partial thromboplastin time (APTT), prothrombin time (PT), thrombin time (TT), and FIB level in plasma was determined using a coagulometer (LG-PABER-I, STEELLEX & Co, Beijing, China), which was operated according to manufacturer instructions. For the positive control, the equivalent amount of Velefibrinase was replaced with urokinase, and for the placebo control, the enzyme was used instead of isotonic saline.

### 2.8. Determination of Hemolysis and Hemorrhagic Activity

The hemolysis activity of the enzyme in vitro was evaluated [27]. In brief, fresh buffalo blood was collected in 3.8% tri-sodium citrate (9:1, *v*/*v*) and washed twice with physiological saline, and diluted to 5% (*v*/*v*) erythrocyte suspension. Aliquots of 1.0 mL erythrocyte suspension were incubated with graded concentrations of the enzyme (0.4–1.2 μM) at 37 °C for 1 h. The morphology of erythrocytes from each group was observed under an optical microscope (COVER-015, OLUMPUS, Tokyo, Japan). The reaction was stopped using an ice bath. After centrifugation at 10,000 rpm at 4 °C for 5 min, the absorbance of the supernatant was recorded at 540 nm. Total hemolysis was achieved by adding 1% Triton X-100 to the erythrocyte suspension. For the control, the enzyme was replaced with isotonic saline.

We also determined the hemorrhagic activity of the enzyme in vivo [28]. Groups of three specific pathogen free (SPF) male BALB/c mice (n = 3 per group) were injected intradermally with different concentrations of Velefibrinase (0.5 and 1.0 mg/kg) in 100 μL of isotonic saline. The group receiving isotonic saline alone served as a control. After 2 h, all animals were euthanized by an overdose of urethane. The dorsal patch of the skin surface was carefully removed without stretching, and the bleeding spot was observed and photographed.

### 2.9. The Effect of Mouse Tail Thrombosis Model of Velefibrinase

The SPF male BALB/c mice (20–30 g) were purchased from Guangxi Medical University Laboratory Animal Center. All experimental protocols were performed according to the “Principles of Laboratory Animal care” (NIH publication no. 8023, revised 1978) and approved by the Animal Ethics Committee of Guangxi University (Permission no.: GXU-2021-046).

The antithrombotic potency of Velefibrinase in vivo was studied in a κ-carrageenan-induced mouse tail thrombosis model. Briefly, mice were divided into six groups (*n* = 6 per group): sham group (isotonic saline, i.v.), model (40 mg/kg κ-carrageenan, i.v.) group, model + urokinase (0.44 mg/kg, i.v.) group, model + low-dosage Velefibrinase (0.22 mg/kg, i.v.), model + middle-dosage (0.44 mg/kg, i.v.), and model + high-dosage (0.88 mg/kg, i.v.). The procedure of the induced mouse tail thrombosis model followed the method of Ma [29]. The length of the wine-colored thrombus infarction in the tail was measured after 24 h.

### 2.10. Statistical Analysis

All experiments were conducted in triplicate and all data are expressed as mean ± standard deviations (SDs). The results were analyzed using a one-way analysis of variance (ANOVA) or Student’s t-test, and *p*-values less than 0.05 was considered statistically significant.

## 3. Results

### 3.1. Screening and Identification of Fibrinolytic Enzyme-Producing Bacterial Strain

The bacterial colonies with a distinct hydrolytic circle around the colony on the fibrin agar plate were chosen for further study. Among the chosen bacteria, the cell-free fermentation supernatant isolated from Z01 exhibited the highest ratio of F/C. Therefore, this bacterium (Z01) demonstrated potential fibrinolytic protease production and was selected for further investigation.

**Figure 1:**
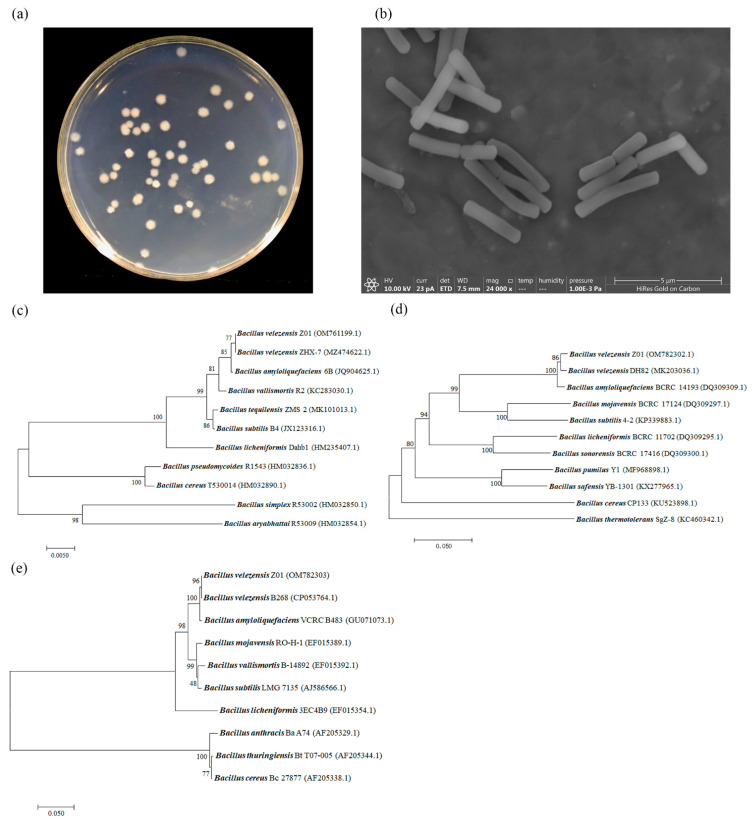
Taxonomic identification of marine microorganism Z01. (**a**) The colony of *B. velezensis* Z01 cultured on a Luria-Bertani plate. (**b**) The *B. velezensis* Z01 was observed under the scanning electron microscope (SEM) with a magnification of 24,000×. Phylogenetic relationships of *B. velezensis* Z01 based on (**c**) partial 16S rDNA sequences, (**d**) *gyrB* and (**e**) *rpoB*.

The strain Z01 on the Luria-Bertani plate was white, round, compressed, and had a flexuous brim (Figure 1a). Under the SEM, the Z01 was rod shaped, with a size of approximately 1.76–4.17 μm (average: 2.83 ± 0.82 μm) in length and 0.47–0.63 μm (average: 0.59 ± 0.05 μm) in width (Figure 1b).

Based on the biochemical properties, strain Z01 was preliminarily identified as the *Bacillus* genus (Supplementary Table S1). Moreover, in terms of 16S partial rDNA (GenBank No. OM761199, 1447 bp), *gyrB* (GenBank No. OM782302, 1160 bp) and *rpoB* (GenBank No. OM782303, 556 bp) sequences of strain Z01 exhibited similarities with those of *Bacillus velezensis* according to the Nucleotide BLAST program and neighbor-joining analysis of MEGA 7 (Figure 1c–e). Thus, we determined that this strain belonged to *Bacillus velezensis*.

### 3.2. Purification and Identification of Velefibrinase

**Figure 2:**
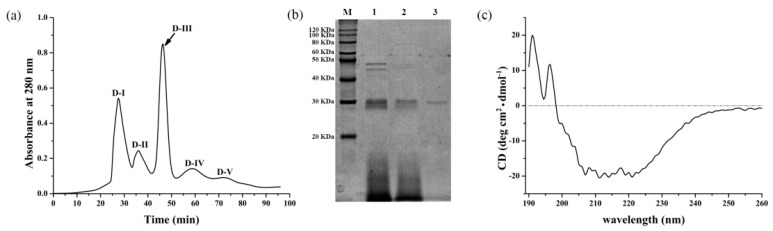
Purification of the fibrinolytic enzyme (Velefibrinase) from *B. velezensis* Z01. (**a**) Purification of Velefibrinase from *B. velezensis* strain Z01 by DEAE-Sephadex chromatography. The eluted proteins were monitored at 280 nm. The arrow indicates eluted Velefibrinase. (**b**) Determination of the purity and molecular mass of Velefibrinase using 12% SDS-PAGE. Lane M, protein molecular mass markers; Lane 1, ammonium sulphate precipitated proteins; Lane 2, HIC-Ⅱ fraction; Lane 3, D-3 fraction. (**c**) The circular dichroism (CD) spectrum of natural Velefibrinase in 0.1 M PBS (pH 7.2).

The crude extract, which was fractionated by 60–80% of precipitated ethanol, was loaded onto a t-Butyl HIC column, which resulted in the separation of proteins into three peaks, designated as HIC-Ⅰ to HIC-Ⅲ (Supplementary Figure S1). The fraction from the un-bound peak (HIC-Ⅰ) and the last retained peak (HIC-Ⅲ) showed no fibrinolytic activity, whereas the second peak (HIC-Ⅱ) exhibited strong fibrinolytic activity. The fraction of HIC-Ⅱ was collected, dialyzed, and concentrated to obtain approximately 3040.82 μg of activity crude enzyme. Then the crude enzyme was applied to a DEAE-Sephadex column, which resolved the five peaks (D-Ⅰ to D-Ⅴ). The D-Ⅲ peak eluted from the retention time of 43 to 52 min showed the highest fibrinolytic activity (Figure 2a). As shown in Table 1, the purification fold and recovery of the purified enzyme were 7.77 and 6.58%, respectively. Finally, the purified enzyme (Velefibrinase) was subjected to 12% SDS-PAGE. The SDS-PAGE profile of Velefibrinase possessed a single band and the molecular weight was approximately 30 KDa (Figure 2b).

According to the MALDI-TOF-MS analysis (Supplementary Figure S2), the molecular weight of Velefibrinase was 323041.47 Da. The matching analysis of the trypsin-digested peptide sequences of Velefibrinase against the UniProt databases of *B. velezensis* (Proteome ID: UP000001120) confirmed two trypsin-digested peptide sequences: (a) VAVIDSGIDSSHPDLK (1652.85 Da) and (b) YPSVIAVGAVNSSNQR (1661.86 Da). This indicated that Velefibrinase belonged to the S8 family protease OS (accession no. tr|A7Z338|A7Z338_BACVZ). Moreover, Velefibrinase, belonging to peptidase S8 A family, was closely related to subtilisin BPN (GenBanK No. P00782) according to the phylogenetic analysis. The predicted amino acid sequence of Velefibrinase shared a sequence identity of 97.38% with subtilisin BPN, 85.30% with subtilisin E (GenBank No. P04189), 85.56% with subtilisin NAT (GenBank No. P35835), and 62.53% with subtilisin A (GenBank No. 00780). The structure of the native Velefibrinase was analyzed using the CD spectrum (Figure 2c). The spectrum revealed two positive peaks at 192 nm and 196 nm and two negative peaks at 194 nm and 221 nm. The secondary structure of Velefibrinase was analyzed through CDPRO, and showed that Velefibrinase consisted of 30.4% alpha-helix, 22.0% beta-sheets, 17.9% beta-turn, and 29.7% random coils.

### 3.3. The Biochemical Properties of Velefibrinase

**Figure 3:**
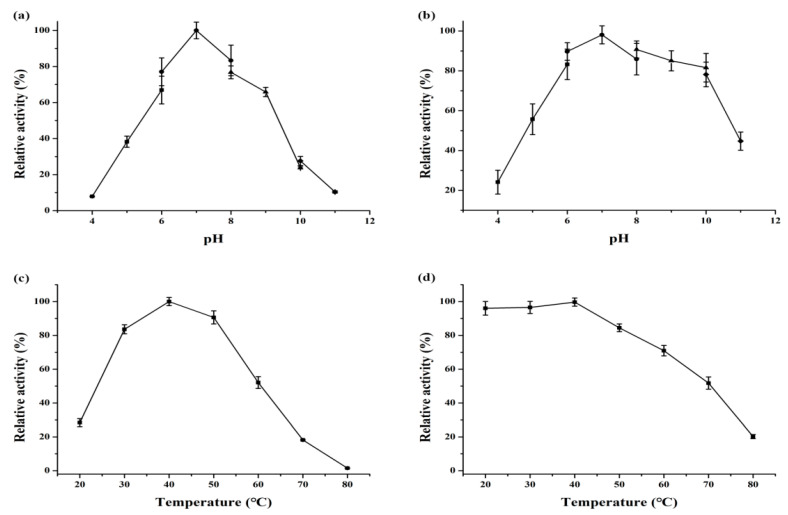
Effects of pH and temperature on fibrinolytic activity and stability. (**a**) The optimum pH of Velefibrinase was determined at a pH range of 3.0 to 13.0. The maximum activity was observed at pH 7.0 and was considered to be 100%. (**b**) After incubation for 24 h, the residual protease activity was measured to investigate the pH stability of Velefibrinase. The enzyme activity before incubation was regarded as 100%. (**c**) The optimum temperature of Velefibrinase was estimated at a temperature range of 20–80 °C. The maximum activity was obtained at 40 °C and was considered to be 100%. (**d**) The thermal stability of Velefibrinase was evaluated after incubation in 0.02 M PBS (pH 7.0) at various temperatures for 24 h.

The effect of pH on the enzyme activity of Velefibrinase was assayed at a pH range of 4.0 to 11.0, and the results are shown in Figure 3a. The Velefibrinase showed fibrinolytic activity at a pH value of 5.0 to 10.0, and the optimum activity was observed at pH 7.0. The effect of pH on Velefibrinase stability is presented in Figure 3b. When the pH was in the range of 6.0–10.0, the enzyme maintained at least 75% of the maximum activity. As shown in Figure 3c, Velefibrinase exhibited activity at 30–60 °C and maximal activity at 40 °C. Moreover, the thermal stability of Velefibrinase was determined for 20 °C to 80 °C, the results are shown in Figure 3d. More than 70% of its maximal activity was retained between 20 °C and 60 °C.

The effect of metal ions and inhibitors on Velefibrinase is presented in Table 2. For the metal ions with a concentration of 5 mM, Mg^2+^ and Ca^2+^ slightly enhanced fibrinolytic activity by 4.37% and 3.25%, respectively, but these were not significantly different from the control. Moreover, Mn^2+^ inhibited the activity of Velefibrinase by 10.61% (*p* > 0.05), whereas the metal ions of Zn^2+^ and Co^+^ significantly inhibited enzyme activity by 56.68% and 48.01% (*p* < 0.01), respectively. Finally, Cu^2+^ and Fe^3+^ completely inhibited the activity of Velefibrinase. For the protease inhibitor, pepstatin A (a pepsin inhibitor) slightly, albeit not significantly, inhibited the activity of Velefibrinase by 3.84%. However, β-mercaptoethanol (a disulfide bond reducing agent), PMSF (a serine protease inhibitor), and EDTA (a metalloprotease inhibitor) significantly inhibited enzyme activity by 10.06% (*p* < 0.05), 93.54% (*p* < 0.01) and 81.62% (*p* < 0.01), respectively.

The substrate specificities of Velefibrinase are listed in Table 3. The enzyme preferred to hydrolyze fibrin the most, followed by FIB and casein. However, Velefibrinase had an insufficient capacity to hydrolyze serum albumin. The calculation using the Lineweaver-Burk double reciprocal plot (Supplementary Figure S3) resulted in *K*_m_ and *V*_max_ values for the fibrinogenolytic activity of Velefibrinase of 18.16 μmol/L and 59.52 μmol/min/mg of protein, respectively. In addition, for fibrinolytic activity, the values of *K*_m_ and *V*_max_ were 8.57 μmol/L and 63.69 μmol/min/mg of protein, respectively.

### 3.4. Fibrin(ogen)olytic and Thrombolysis Ability of Velefibrinase

To investigate whether Velefibrinase could convert plasminogen into plasmin, the fibrinolytic activity of the enzyme was determined using both plasminogen-free and plasminogen-rich fibrin plates. Results showed that the transparent circle area in the plasminogen-rich fibrin plate was 28.12% larger than that in the plasminogen-free fibrin plate, which suggested that Velefibrinase possessed kinase activity and the ability to convert plasminogen into plasmin (Supplementary Figure S4).

**Figure 4:**
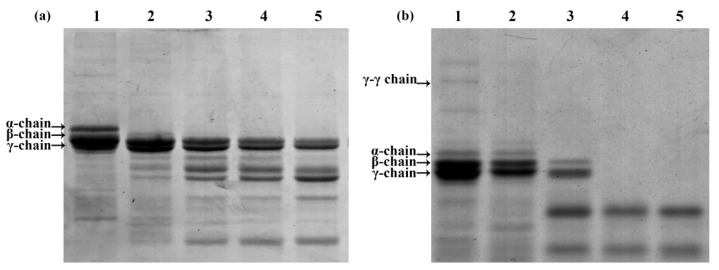
Determination of time-dependent degradation of fibrin and fibrinogen (FIB) of Velefibrinase. The degradation pattern of (**a**) FIB and (**b**) fibrin by Velefibrinase is shown using 12% SDS-PAGE. Lanes 1–5 are degradation products incubated (0.02 M PBS, pH 7.2, 37 °C) after of 0, 20, 40, 60, and 80 min, respectively.

To elucidate the degradation pattern of fibrin/FIB, we analyzed the enzyme using 12% SDS-PAGE. As shown in Figure 4a, FIB was divided into Aα, Bβ, and γ chains. According to the gray scanning analysis, when incubated with Velefibrinase, the Aα chain was rapidly digested. Then, the Bβ chain was digested slightly, whereas the γ chain showed little change. The degradation pattern of fibrin by Velefibrinase was also analyzed (Figure 4b), and results showed that Velefibrinase completely degraded fibrin within 60 min. The γ-γ chain was first hydrolyzed within 20 min, the α chain of fibrin was hydrolyzed within 40 min, and finally, the β and γ chains were degraded at 60 min of incubation.

In vitro, the thrombolysis ability of Velefibrinase was evaluated with clots from buffalo whole blood (Table 4). Velefibrinase displayed thrombolysis activity on the blood clots in a dose-dependent manner. When the concentration of Velefibrinase was 0.2 μM, the blood clots lysis rate was higher than 50%, and the clots could be completely dissolved at concentrations of up to 0.8 μM. Moreover, the clot lysis rate differed significantly between each group (*p* < 0.05).

### 3.5. Antiplatelet and Anticoagulation of Velefibrinase

**Figure 5:**
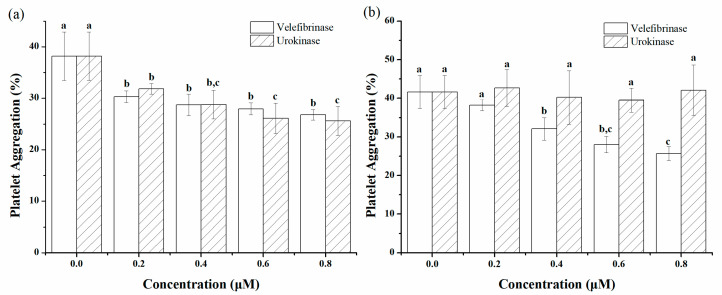
Determination of the platelet aggregation rate induced by adenosine diphosphate (ADP) in vitro. The effect of Velefibrinase and urokinase (0–0.8 μM) on platelet aggregation in (**a**) platelet-rich plasma (PRP) or (**b**) wash platelet (WP). Data represent mean ± standard deviations (SDs) of triplicate experiments. Different letters indicate significant differences (*p* < 0.05) in each group.

In PRP and WP, 5 μM of ADP successfully induced platelet aggregation, and the aggregation rate was 38.20% and 41.63%, respectively. The results of the antiplatelet aggregation property of Velefibrinase and urokinase in PRP are shown in Figure 5a. About 0.2 μM of Velefibrinase and urokinase significantly reduced the platelet aggregation rate by 20.68% (*p* < 0.01) and 16.68% (*p* < 0.05), respectively, compared to the control (0 μM). With the increase of concentration, Velefibrinase (0.2–0.8 μM) could continue to reduce the platelet aggregation rate, but there was no significant difference (*p* > 0.05) for each groups. However, 0.6 μM of urokinase continuously significantly inhibited platelet aggregation (*p* < 0.05). As shown in Figure 5b, Velefibrinase significantly inhibited platelet aggregation in WP at 0.4 μM (*p* < 0.01), which reduced the aggregation rate to 32.07%. Moreover, 0.8 μM of Velefibrinase significantly reduced the platelet aggregation by 19.96% (*p* < 0.05) compared to that of 0.4 μM, and the inhibitory effect was better than that of PRP. In contrast, urokinase (0.2–0.8 μM) did not exhibit antiplatelet aggregation in WP.

**Figure 6:**
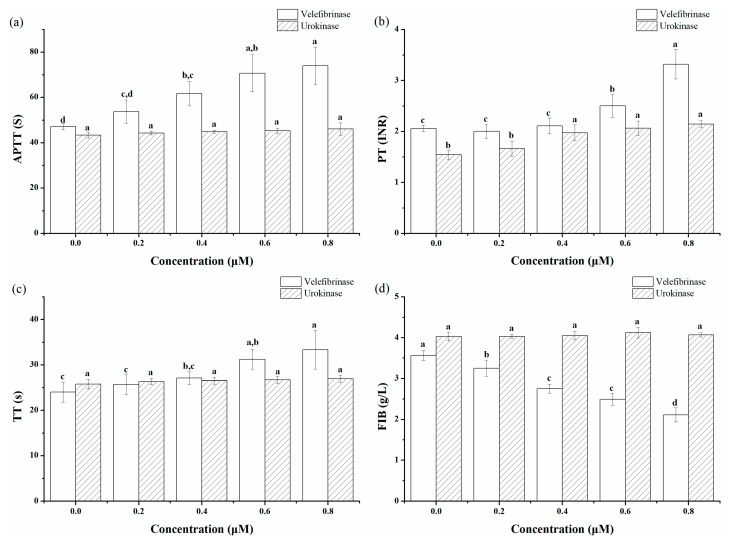
Determination of the anticoagulant activity in vitro. The effect of Velefibrinase and urokinase (0–0.8 μM) on (**a**) activated partial thromboplastin time (APTT), (**b**) prothrombin time (PT), (**c**) thrombin time (TT), and (**d**) fibrinogen (FIB) in PPP. Data represent mean ± standard deviations (SDs) of triplicate experiments. Different letters indicate significant differences (*p* < 0.05) in each group.

Figure 6 shows the comparison between the anticoagulation potency of Velefibrinase and that of the commercial anticoagulant urokinase. In the control group (0 μM), APTT, PT, TT, and FIB were 47.10 s, 2.06, 24.03 s, and 3.57 g/L, respectively. Velefibrinase affected APTT, PT, TT, and FIB in a dose-dependent manner. A total of 0.2 μM of Velefibrinase significantly reduced the content of FIB in plasma to 3.25 g/L (*p* < 0.05); APTT and TT increased, but not significant difference was found; the PT level was similar to that of the control group. Furthermore, 0.4 μM of Velefibrinase significantly increased the APTT by 31.00% (*p* < 0.05) compared to that of the control group; and the content of FIB was further significantly reduced to 2.75 g/L (*p* < 0.01). About 0.6 μM of Velefibrinase significantly prolonged TT and PT by 30.12% (*p* < 0.01) and 21.36% (*p* < 0.05), respectively, compared to that of the control group. Although urokinase (0.2–0.8 μM) slightly prolonged APTT and TT, the change was not significant; moreover, it had almost no effect on the content of FIB in plasma, and only significantly prolonged PT by 27.87% at 0.4 μM (*p* < 0.01).

### 3.6. Hemolytic and Hemorrhagic Activity of Velefibrinase

We evaluated the hemolytic activity for erythrocyte in vitro and the hemorrhagic activity of Velefibrinase in vivo (Supplementary Figure S5). When the concentration of Velefibrinase was as higher as 1.2 μM, the erythrocytes maintained a normal morphology and did not exhibit the distinct erythrocyte ghost phenomenon under a microscope. The maximum hemolysis of mammalian wash erythrocytes of Velefibrinase was only 4.82%, which was not significantly different from that of the isotonic saline control (Supplementary Table S2). Moreover, the Velefibrinase (0.5 and 1.0 mg/kg) did not induce hemorrhaging in mice when administered intradermally.

### 3.7. Evaluation of Therapeutic Efficacy In Vivo

**Figure 7:**
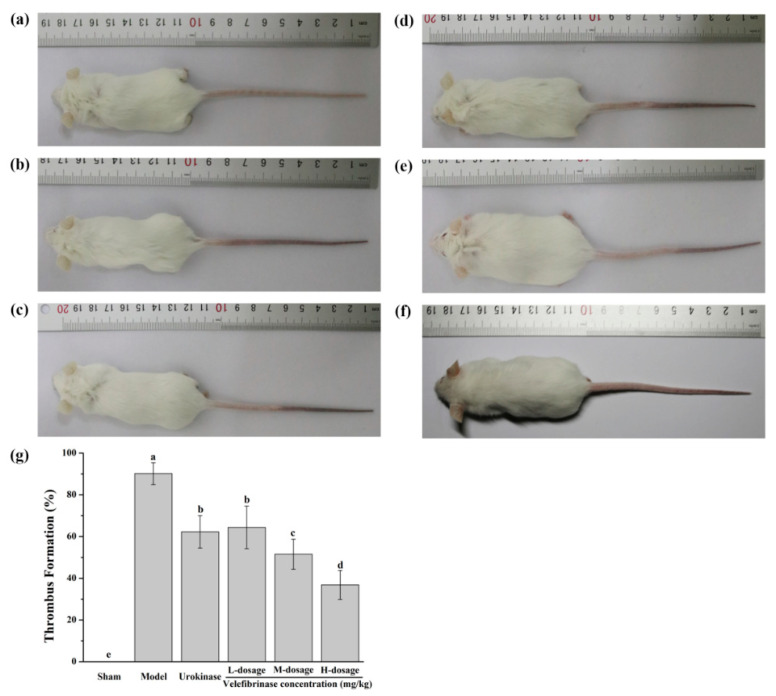
Velefibrinase ameliorated the length of the mouse tail thrombus induced by κ-carrageenan (*n* = 6 per group). The mice were injected intravenously (**a**) isotonic saline only (sham), (**b**) 40 mg/kg κ-carrageenan (model), (**c**) 40 mg/kg κ-carrageenan + 0.44 mg/kg urokinase, (**d**) 40 mg/kg κ-carrageenan + 0.22 mg/kg Velefibrinase (low-dosage), (**e**) 40 mg/kg κ-carrageenan + 0.44 mg/kg Velefibrinase (middle-dosage), and (**f**) 40 mg/kg κ-carrageenan + 0.88 mg/kg Velefibrinase (high-dosage). (**g**) The length of the mouse tail infarcted region of different treatment groups. Each value is expressed as a mean ± standard deviations (SDs). Different letters indicate significant differences (*p* < 0.05) in each group.

In this study, the antithrombotic ability of Velefibrinase was evaluated in a κ-carrageenan-induced mouse tail thrombosis model. The effect of Velefibrinase on a mouse tail thrombus induced by κ-carrageenan is shown in Figure 7. In the sham group, there was no thrombus formation in the mouse tails. When mice were injected with κ-carrageenan after 24 h, the average length of the tail infarction region in the thrombus model group was 83.56 mm, of which the infarction region accounted for 90.17% of the total length of the tail. Compared with the model group, the infarction region in low-dosage (0.22 mg/kg) Velefibrinase group was significantly ameliorated by 25.57% (*p* < 0.01). The thrombus infarction region would be further decreased with the increase in Velefibrinase concentration, which indicated that Velefibrinase could ameliorate the mice tail thrombus infarction region in a dose-dependent manner. When the concentration of Velefibrinase was as higher as 0.8 mg/kg (high-dosage), the tail thrombus region decreased to 36.82%. Urokinase (0.44 mg/kg) significantly reduced the length of the thrombus infarction region (62.12%, *p* < 0.01), but the therapeutic efficacy did no differ significantly from that of the low-dosage Velefibrinase group, and was significantly weaker than that of the equal-dose Velefibrinase group (*p* < 0.05).

## 4. Discussion

In this study, a strain that produce fibrinolytic enzyme was screened from marine mud of the South China Sea. By sequencing and conducting blast alignment of three conserved genes, 16 rDNA, *gyrB,* and *rpoB*, combined with physiological and phenotypic characteristics analysis, it was confirmed that the strain belonged to *B. velezensis* named Z01. *B. velezensis* has been widely used in the agro-industrial field, such as for biological control and yield improvement [14]. Moreover, Ye et al. [30] reported that the use of *B. velezensis* as a feed additive to replace feed antibiotics enhanced production performance and egg quality and demonstrated its potential as a probiotic. However, there are a few reports on fibrinolytic protease from *B. velezensis*.

Velefibrinase (a fibrinolytic protease) was purified to homogeneity following four steps, with a 6.58% recovery and a 7.77-fold increase in specific activity. It is worth noting that a large amount of enzyme activity was lost during the alcohol precipitation step, which may have been caused by the limited range of alcohol concentrations in alcohol precipitation. Nevertheless, this selection was beneficial for the removal of impurities and subsequent purification. According to SDS-PAGE and MALDI-TOF-MS, the molecular mass of Velefibrinase was approximately 32.3 KDa, which was comparable to the molecular mass of other fibrinolytic enzymes (18–38 KDa) isolated from other genera of *Bacillus* [31]. The molecular mass of Velefibrinase was lower than that of many other fibrinolytic enzymes, such as Brevithrombolase (56 KDa) [32] and Russelobin (51.3 KDa) [27], but also higher than that of several fibrinolytic enzymes such as PAIEM (14.9 KDa) [33] and C142 (23.5 KDa) [34]. Moreover, Velefibrinase belonged to the peptidase S8 family (subtilisin family, clan SB), which is similar to other fibrinolytic enzymes purified from *B. velezensis* SW5 [18].

Velefibrinase maintained fibrinolytic activity across a broad range of pHs and temperatures, with an optimum pH and temperature 7.0 and 40 °C, which is in line with the physiological environment of the human body. This finding is the same as that of other fibrinolytic enzymes from *Streptomyces radiopugnans* VITSD8 [35] and *Serratia marcescens* [36], which are all derived from the marine environment. In addition, Velefibrinase became unstable at temperatures above 70 °C, which verified that the enzyme had good thermal stability. In the CD spectrum, Velefibrinase showed a depth negative ellipticity at 221 nm, which can explain the good thermal stability [20].

Inhibition studies can offer valuable insight into the nature of the active center of enzymes [37]. Because PMSF and EDTA strongly inhibited the fibrinolytic activity of Velefibrinase, this suggested that Velefibrinase is a serine and metalloprotease enzyme. Moreover, the inhibition of enzyme activity in the presence of a β-mercaptoethanol environment indicated that intramolecular disulfide bonds may exist in protease. Generally, metal cations play an essential role in the regulation of enzyme activity. In this study, Cu^2+^, Zn^2+^, Fe^3+^, and Co^+^ significantly inhibited enzyme activity, which reveal that these cations intrude and bind to carboxyl groups that may be an essential component of the active center of the enzyme. In contrast, Ca^2+^ and Mg^2+^ significantly improve fibrinolytic activity, which is similar to the novel fibrinolytic enzyme isolated from *Lyophyllum shimeji* [38].

Fibrinolytic enzymes are usually classified into α, β, and α/β fibrinogenases on the basis of degradation of Aα-chain or Bβ-chain of FIB [37]. Based on degradation pattern of fibrin and FIB, Velefibrinase should be classified as α/β-fibrinogenase. On the one hand, the *K*_m_ of Velefibrinase to fibrin was 8.57 μmol/L (2.91 g/L), which was lower than that of Bvsp (3.19 g/L) which was from a marine bacterium *B. vallismortis*, and JP-Ⅰ (0.43 mmol/L), Ⅱ (0.69 mmol/L) which was from a Korean traditional fermented food, but higher than that of lumbrokinase (2.67 g/L) [39,40]. On the other hand, the *K*_m_ of Velefibrinase to FIB was 18.16 μmol/L (6.17 g/L), which was higher than FIB content in human plasma (2.4–5.0 g/L) and that of nattokinase (2.19 g/L) [2]. Therefore, Velefibrinase preferentially degrades fibrin and may not induce side effects, such as excessive bleeding. A higher F/C ratio indicated that fibrin could be degraded efficiently and quickly [37]. Moreover, the F/C ratio of Velefibrinase (1.48) was higher than that of human plasmin (1.33) and other bacterial fibrinolytic enzymes, such as FCF-11 (0.71), Subtilisin BPN (0.32), Subtilisin Carlsberg (0.92), protease from *B. licheniformis* (0.93) and fibrinolytic serine metalloprotease from marine *S. marcescens subsp. sakuensis* (0.31) [36,41,42]. However, it has also been reported that the F/C ratio of Velefibrinase is lower than that of Brevithrombolase (2260.0) [32] and Bacethrombase (24.0) [43]. Furthermore, Velefibrinase converts plasminogen into plasmin, which further demonstrates its efficient thrombolytic ability. In summary, these findings indicate that Velefibrinase has high fibrinolytic efficiency to degrade the thrombus.

Platelet aggregation plays an essential role in physiological hemostasis and thrombosis [44]. Velefibrinase inhibits platelet aggregation; however, its anti-platelet aggregation ability is lower than that of other fibrinolytic enzymes [43]. Conversely, this mild inhibition of platelet aggregation can reduce the risk of bleeding, which is more conducive to clinical applications. The anti-platelet aggregation activity of Velefibrinase in PRP was lower than that in WP, which suggested that Velefibrinase acts on other proteins in plasma. Thus, this influences the anti-platelet ability of Velefibrinase in PRP. Moreover, we noted that Velefibrinase inhibited platelet aggregation in WP. Because Velefibrinase is a macromolecule, it is unlikely to interact with intercellular factors in signaling pathways. Therefore, Velefibrinase may act directly on platelet membrane receptors and/or integrins to inhibit platelet aggregation. In addition to the inhibition of platelet aggregation, Velefibrinase may also intervene with coagulation cascades. The blood coagulation cascade is divided into intrinsic, extrinsic, and common pathways. APTT was used to evaluate the factors in the intrinsic and common pathways of blood coagulation, such as factors VIII, IX, XI, and vWF, whereas PT was used to assess extrinsic coagulation factors, such as factors V, VII, and X. TT reflected the fibrin polymerization process and was considered the third coagulation phase, together with FIB [26]. These results demonstrated that the anticoagulant action of Velefibrinase during dose-dependent treatment is modified by both intrinsic and extrinsic pathways of the blood coagulation cascade.

To preliminarily investigate the safety of Velefibrinase as a clinical application, we carried out hemolysis and hemorrhagic activity tests. They indicated that Velefibrinase exhibited no hemolysis for erythrocyte in vitro and no hemorrhagic activity in vivo. This result is similar to other fibrinolytic enzymes from *Bacillus*, which has a good safety profile [32,37,43]. Moreover, the thrombolytic activity of Velefibrinase in vivo was measured in a κ-carrageenan-induced tail thrombus model, which has the major advantages of visibility of the thrombus infraction region and accuracy [45]. Results indicated that the Velefibrinase significantly ameliorated the length of the tail thrombus, and the therapeutic efficacy significantly surpassed that of an equal dose of urokinase. Moreover, the thrombolytic activity of Velefibrinase in vivo was found to be superior to that of Subtilisin QK [45], DEF [46], u-PA [47], and lumbrokinase [48], but inferior to that of streptokinase [43]. Therefore, this suggested that Velefibrinase is a safe thrombolytic or blood-thinning agent for clinical applications in patients with hyperfibrinogenemia and associated thrombolytic disorders.

## 5. Conclusions

A fibrinolytic enzyme (Velefibrinase) found in marine *B.* *velezensis* Z01 showed no hemolysis and hemorrhagic activity and is a serine metalloprotease, which exhibits anticoagulation and antiplatelet activity. Purified Velefibrinase with 32.3 KDa had high substrate specificity to fibrin and was classified as an α/β-fibrinogenase. In vitro, Velefibrinase exhibited strong thrombus-dissolving efficacy, and anti-platelet activity and ameliorated blood coagulation. Moreover, the thrombolytic effect of Velefibrinase was superior to that of commercial thrombolytic drug in vivo. Therefore, Velefibrinase may be a promising candidate as an effective thrombolytic agent in the clinic.

## Figures and Tables

**Table 1:** Steps of the purification process of Velefibrinase from *B. velezensis Z01*.

Purification Step	Total Protein (μg)	Total Activity (U) ^a^	Specific Activity (U/μg)	Purification Fold	Recovery (%)
Crude extract	41,863.73	411,967.12	9.84	1.00	100.00
Ethanol precipitation (60/80%)	7737.23	114,319.23	14.76	1.50	27.75
t-Butyl HIC	3040.82	85,349.13	28.07	2.85	20.72
DEAE-Sephadex	354.65	27,115.40	76.46	7.77	6.58

^a^ The activity was measured by the fibrin plate assay. The units of activity are calculated based on the urokinase standard.

**Table 2:** Effect of metal ions and protease inhibitors on the activity of Velefibrinase.

Metal Ions and Protease Inhibitors	Concentration (mM)	Relative Activity (%)
Control	--	100
Cu^2+^	5	-- **
Zn^2+^	5	43.32 ± 1.32 **
Mg^2+^	5	104.37 ± 5.14
Ca^2+^	5	103.25 ± 4.08
Fe^3+^	5	-- **
Mn^2+^	5	89.39 ± 3.23
Co^+^	5	51.99 ± 4.56 **
PMSF	5	6.46 ± 1.71 **
EDTA	5	18.38 ± 2.06 **
β-Mercaptoethanol	5	89.94 ± 1.46 *
Pepstatin A	1 × 10^−3^	96.16 ± 5.12

Values represent mean ± SD of triplicate determinations. --: The enzyme activity was totally inhibited. Significance difference with respect to control: * *p* < 0.05, ** *p* < 0.01.

**Table 3:** Determination of substrate specificity of Velefibrinase.

Substrate	Activity (U/mg) ^a^
Fibrin	61.30 ± 2.45
Fibrinogen	45.82 ± 1.91
Casein	41.29 ± 2.61
Serum albumin	7.61 ± 0.60

Values represent mean ± standard deviations (SDs) of triplicate determinations. ^a^ Unit is defined as 1.0 μg of tyrosine equivalent liberated per min per mL after 30 min incubation at 40 °C, pH 7.0.

**Table 4:** The effect of Velefibrinase on the lysis of blood clots.

The Concentration (μM)	Blood Clot Lysis Rate (%)
0 (Control)	0 ± 3.83 ^a^
0.2	51.88 ± 4.75 ^b^
0.4	73.64 ± 5.47 ^c^
0.6	90.38 ± 1.92 ^d^
0.8	Complete lysis ^f^

All values are mean ± standard deviations (SDs) of three independent experiments. The lysis rate of blood clots was assessed at 37 °C for 10 h. Different letters indicate significant differences (*p* < 0.05) in each group.

## Data Availability

All data underlying the results are included as part of the published article.

## Supplementary Materials

The following supporting information can be downloaded at: https://www.mdpi.com/article/10.3390/microorganisms10050843/s1. Figure S1: Purification of Velefibrinase from B. velezensis strain Z01 by t-Butyl HIC chromatography. The elution of proteins was monitored at 280 nm. The peak of HIC-Ⅱ with fibrinolytic activity. Figure S2: (a) The MALDI-TOF-MS spectrum of trypsin-digested peptide sequences of Velefibrinase. The molecular mass of Velefibrinase was 323041.47 Da. Two of peptide sequences in Velefibrinase were confirmed: VAVIDSGIDSSHPDLK (1652.85 Da) and YPSVIAVGAVNSSNQR (1661.86 Da). (b) Prediction of the conserved domain of Velefibrinase. (c) Phylogenetic analysis of Velefibrinase and some other typical peptidases from the S8 family using the neighbor-joining method. (d) Multiple sequence alignment of Velefibrinase and some other typical peptidases from the S8 family. Figure S3: The kinetics of Velefibrinase on (a) fibrinogenolytic and (b) fibrinolytic activity. The Km and Vmax of Velefibrinase against various concentrations of fibrinogen and fibrin were determined by Lineweaver–Burk plot. Figure S4: The comparison of transparent area of Velefibrinase in (a) plasminogen-rich fibrin plate and (b) plasminogen-free fibrin plate. Figure S5: The hemolytic activity for erythrocyte in vitro and hemorrhagic activity in vivo of Velefibrinase were evaluated. (a) Aliquots of 1.0 mL 5% washed suspension were incubated with different concentrations of Velefibrinase at 37 °C for 1 h. As for negative control, the Velefibrinase was replaced with 1% Triton X-100. The morphology of erythrocytes was observed under an optical microscope (200×). (b) Aliquots of 0.1 mL Velefibrinase with different concentrations were injected intradermally into mice, and the group receiving isotonic saline alone served as control. After 2 h, mice were sacrificed; skin was removed and observed. Table S1: The morphological and physiological characteristics of B. velezensis Z01. Table S2: The effect of different concentrations of Velefibrinase on hemolysis rate of wash erythrocytes from buffalo.
