# Supplementary material for: Purification and Characterization of a Fibrinolytic Enzyme from Marine Bacillus velezensis Z01 and Assessment of Its Therapeutic Efficacy In Vivo
Source: Microorganisms. 2022 Apr 20;10(5):843. doi: 10.3390/microorganisms10050843 (PMC9145925; doi:10.3390/microorganisms10050843)

Supplementary Material

Article title: Purification and characterization of a fibrinolytic enzyme from marine *Bacillus velezensis* Z01 and assessment of its therapeutic efficacy *in vivo*

Journal name: microorganisms

Authors: Yuting Zhou [a], Huizhen Chen [a], Bo Yu [a], Guiguang Chen [a,*], Zhiqun Liang [a,*]

[a] State Key Laboratory for Conservation and Utilization of Subtropical Agro-bioresources, Guangxi Microorganism and Enzyme Research Center of Engineering Technology, College of Life Science and Technology, Guangxi University, 100 Daxue Road, Nanning 530004, Guangxi, P. R. China

***Correspondence author:**

Guiguang Chen and Zhiqun Liang, Post code: 530004. Tel. / fax: +86 0771 3271181.

E-mail address: chgg0313@gxu.edu.cn (G.C.); zqliang@gxu.edu.cn (Z.L)

Legends of Supplementary Figures and Tables

Supplement Table S1. The Morphological and physiological characteristics of *B. velezensis* Z01

Supplement Table S2. The effect of different concentrations of Velefibrinase on hemolysis rate of wash erythrocytes from buffalo

Supplementary Figure S1. Purification of Velefibrinase from *B. velezensis* strain Z01 by t-Butyl HIC chromatography. The elution of proteins was monitored at 280 nm. The peak of HIC-II with fibrinolytic activity.

Supplementary Figure S2. (a) The MALDI-TOF-MS spectrum of trypsin-digested peptide sequences of Velefibrinase. The molecular mass of Velefibrinase was 323041.47 Da. Two of peptide sequences in Velefibrinase were confirmed: VAVIDSGIDSSHPDLK (1652.85 Da) and YPSVIAVGAVNSSNQR (1661.86 Da). (b) Prediction of the conserved domain of Velefibrinase. (c) Phylogenetic analysis of Velefibrinase and some other typical peptidases from the S8 family by using the neighbor-joining method. (d) Multiple sequence alignment of Velefibrinase and some other typical peptidases from the S8 family.

Supplementary Figure S3. The kinetics of Velefibrinase on (a) fibrinogenolytic and (b) fibrinolytic activity. The K_m and V_{max} of Velefibrinase against various concentrations of fibrinogen and fibrin were determined by Lineweaver–Burk plot.

Supplementary Figure S4. The comparison of transparent area of Velefibrinase in (a) plasminogen-rich fibrin plate and (b) plasminogen-free fibrin plate.

Supplementary Figure S5. The hemolytic for erythrocyte *in vitro* and hemorrhagic activity *in vivo* of Velefibrinase was evaluated. (a) An aliquots of 1.0 mL 5% washed suspension were incubated with different concentrations of Velefibrinase at 37 °C for 1 h. As for negative control, the Velefibrinase was replaced by the 1% Triton X-100. The morphology of erythrocytes was observed under the optical microscope (200 ×). (b) An aliquots of 0.1 mL Velefibrinase with different concentrations was injected intradermally into mice, and the group receiving isotonic saline alone served as a control. After 2 h, mice were sacrificed; skin was removed and observed.

Supplement Table S1

Characteristic	Result	Characteristic	Result
Cell morphology	Rod-shaped	Carbohydrate utilization	
Gram staining	+	D-Glucose	+
Form of spores	+	D-Fructose	+
Motility	-	L-Arabinose	+
Catalase activity	+	Mannitol	+
Urease activity	+	Maltose	+
Anaerobic growth	-	Gas from glucose	-
Voges-Proskauer test	+	Growth at 45 ℃	+
Liquification of gelatin	+	Growth at 50 ℃	-
Hydrolysis of starch	+	Growth at pH 5.0	+
Hydrolysis of casein	+	Growth at pH 9.0	+
Production of indole	-	Growth with 5% NaCl	+
Reduction of nitrates	-	Growth with 7% NaCl	+
Citrate Utilization	-	Growth with 12% NaCl	+

+: indicating growth well or positive; -: indicating no growth or negative.

Supplementary Table S2

Concentration (μM)	Hemolysis rate (%)
0 (Isotonic saline)	0 ± 0.57 [b]
0.2	4.55 ± 0.82 [b]
0.4	4.78 ± 0.76 [b]
0.8	4.82 ± 0.53 [b]
1.2	4.77 ± 0.77 [b]
1% Triton X-100	100 ± 3.13 [a]

All values are mean ± SD of three independent experiments. The hemolysis rate was assessed at 37 °C for 1 h. Under the environment of isotonic saline and 1% Triton X-100, the erythrocytes of hemolysis rate was regarded as 0% and 100%, respectively.

Different letters meant the hemolysis rate was a significant difference in each group.

Supplementary Figure S1.

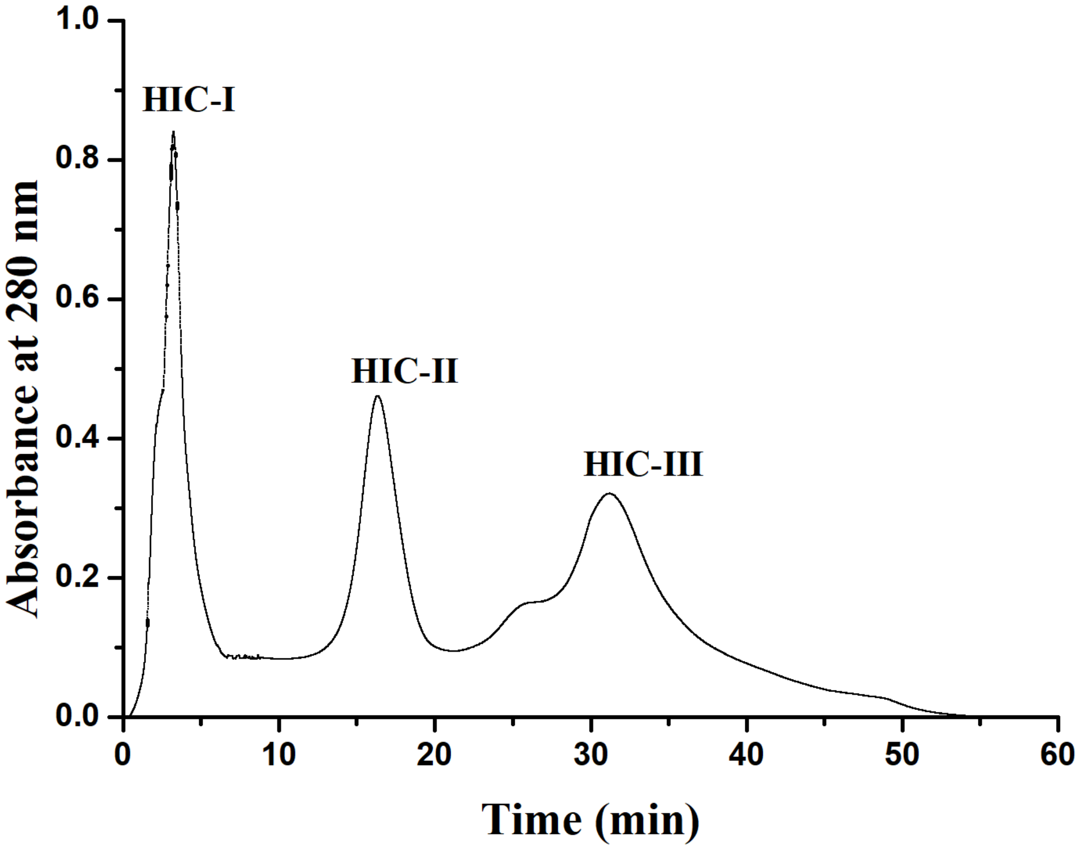

Supplementary Figure S2.

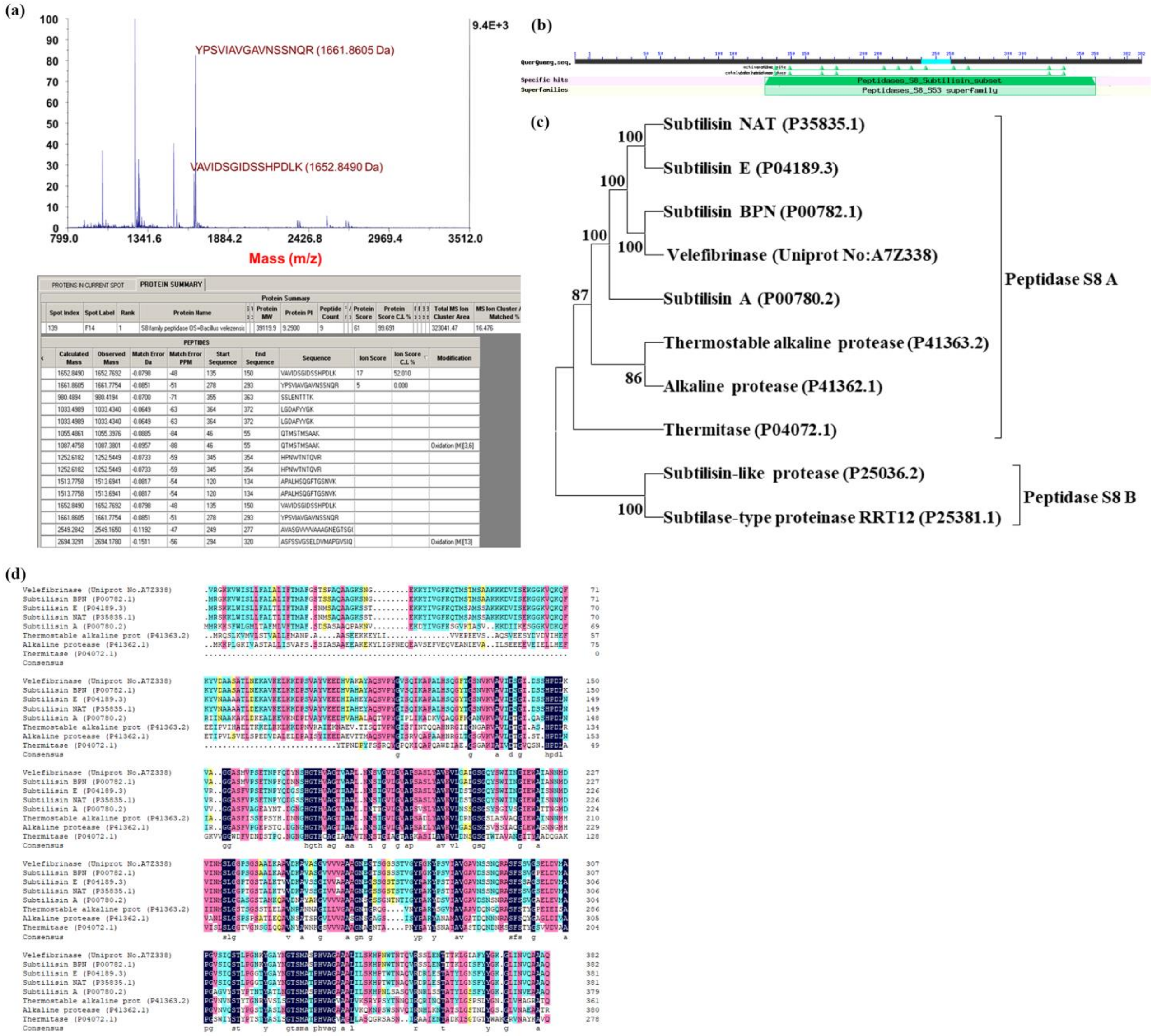

PROTEINS IN CURRENT SPOT | PROTEIN SUMMARY

Protein Summary

Spot Index	Spot Label	Rank	Protein Name	Protein MW	Protein PI	Peptide Count	Protein Score	Protein Score C.I. %	Total MS Ion Cluster Area	MS Ion Cluster Matched %
139	F14	1	S8 family peptidase OS=Bacillus velezensis	39119.9	9.2900	9	61	99.691	323041.47	16.476

PEPTIDES

Calculated Mass	Observed Mass	Match Error Da	Match Error PPM	Start Sequence	End Sequence	Sequence	Ion Score	Ion Score C.I. %	Modification
1652.8490	1652.7692	-0.0798	-48	135	150	VAVIDSGIDSSHPDLK	17	52.010	
1661.8605	1661.7754	-0.0851	-51	278	293	YPSVIAVGAVNSSNQR	5	0.000	
980.4894	980.4194	-0.0700	-71	355	363	SSLENTTTK			
1033.4989	1033.4340	-0.0649	-63	364	372	LGDAFYYGK			
1033.4989	1033.4340	-0.0649	-63	364	372	LGDAFYYGK			
1055.4861	1055.3976	-0.0885	-84	46	55	QTMSTMSAAK			
1087.4758	1087.3801	-0.0957	-88	46	55	QTMSTMSAAK			Oxidation (M)[3,6]
1252.6182	1252.5449	-0.0733	-59	345	354	HPNWTNTQVR			
1252.6182	1252.5449	-0.0733	-59	345	354	HPNWTNTQVR			
1513.7758	1513.6941	-0.0817	-54	120	134	APALHSQGFTGSNVK			
1513.7758	1513.6941	-0.0817	-54	120	134	APALHSQGFTGSNVK			
1652.8490	1652.7692	-0.0798	-48	135	150	VAVIDSGIDSSHPDLK			
1661.8605	1661.7754	-0.0851	-51	278	293	YPSVIAVGAVNSSNQR			
2549.2842	2549.1650	-0.1192	-47	249	277	AVASGVVVVAAAGNEGTSGI			
2694.3291	2694.1780	-0.1511	-56	294	320	ASFSSVGSELDVMAPGVSIQ			Oxidation (M)[13]

Supplementary Figure S3.

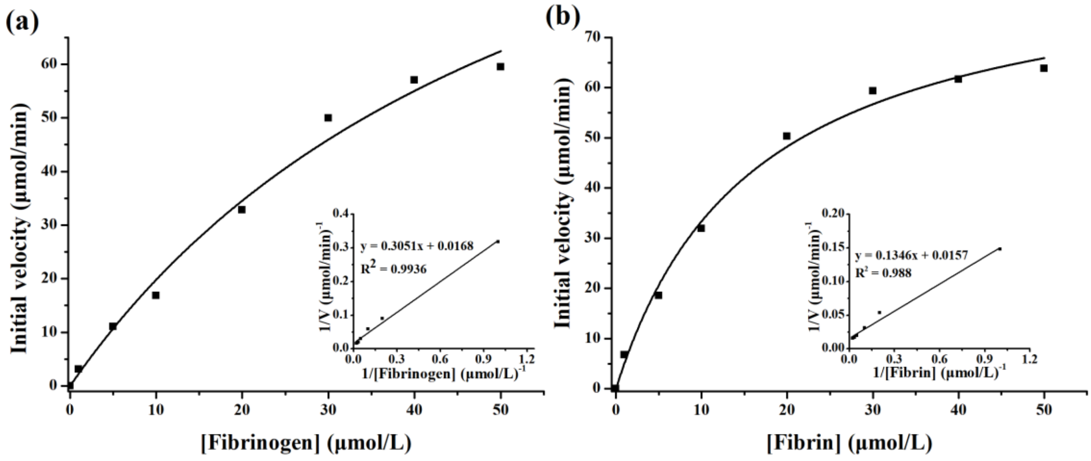

Supplementary Figure S4.

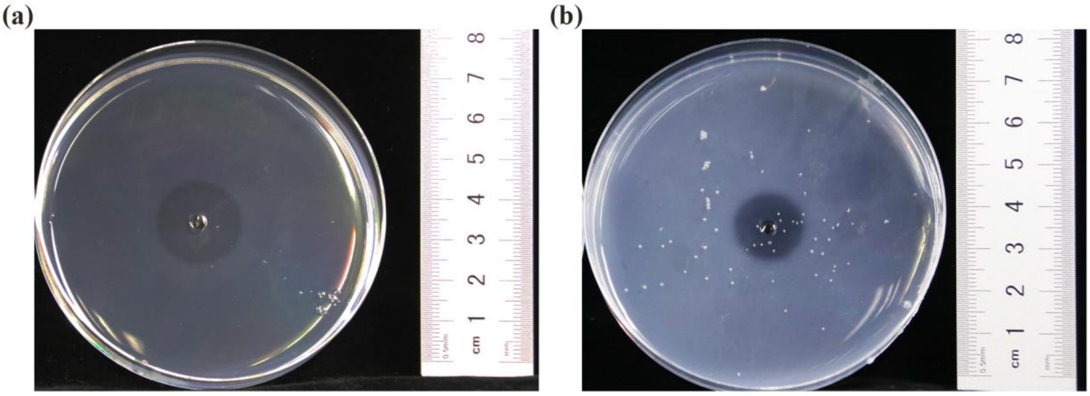

Supplementary Figure S5.

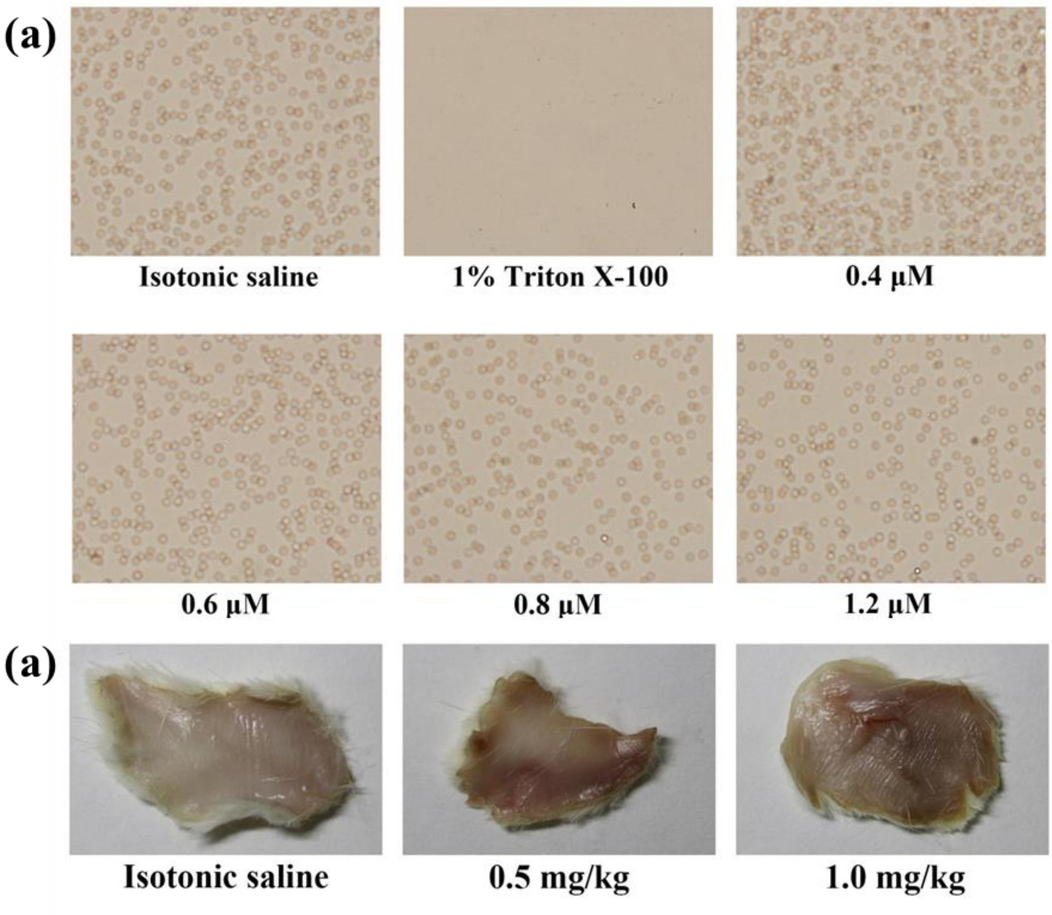

Supplement: Supplementary file 1 [file microorganisms-10-00843-s001.zip › microorganisms-1665759-supplementary.pdf]
